# Supplementary material for: Wx: a neural network-based feature selection algorithm for transcriptomic data
Source: Sci Rep. 2019 Jul 19;9:10500. doi: 10.1038/s41598-019-47016-8 (PMC6642261; doi:10.1038/s41598-019-47016-8)
Supplement: Supplementary file 1 — Supplementary Figure 1 [file 41598_2019_47016_MOESM1_ESM.pdf]

Wx: a neural network-based feature selection algorithm for transcriptomic data

Sungsoo Park[1,†], Bonggun Shin[1,3,†], Won Sang Shim[1], Yoonjung Choi[1], Kilsoo Kang[1], and Keunsoo Kang[2,*]

[1]Deargen Inc., Daejeon, Republic of Korea; [2]Department of Microbiology, College of Natural Sciences, Dankook University, Cheonan 31116, Republic of Korea; [3]Computer Science, Emory University, Atlanta, GA 30322

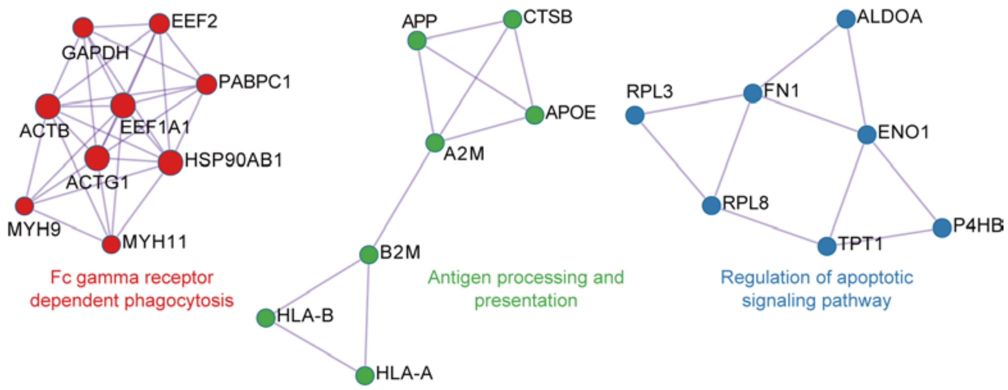

Supplementary Figure 1. Key networks of the top 50 genes with highest *DI* scores are shown.
